# Identification of a novel picornavirus related to cosaviruses in a child with acute diarrhea

**DOI:** 10.1186/1743-422X-5-159

**Published:** 2008-12-22

**Authors:** Lori R Holtz, Stacy R Finkbeiner, Carl D Kirkwood, David Wang

**Affiliations:** 1 Department of Pediatrics, Washington University School of Medicine, St. Louis, MO USA; 2 Departments of Molecular Microbiology and Pathology and Immunology, Washington University School of Medicine, St. Louis, MO USA; 3 Enteric Virus Research Group, Murdoch Childrens Research Institute, Royal Children's Hospital, Victoria, Australia

## Abstract

Diarrhea, the third leading infectious cause of death worldwide, causes approximately 2 million deaths a year. Approximately 40% of these cases are of unknown etiology. We previously developed a metagenomic strategy for identification of novel viruses from diarrhea samples. By applying mass sequencing to a stool sample collected in Melbourne, Australia from a child with acute diarrhea, one 395 bp sequence read was identified that possessed only limited identity to known picornaviruses. This initial fragment shared only 55% amino acid identity to its top BLAST hit, the VP3 protein of Theiler's-like virus, suggesting that a novel picornavirus might be present in this sample. By using a combination of mass sequencing, RT-PCR, 5' RACE and 3' RACE, 6562 bp of the viral genome was sequenced, which includes the entire putative polyprotein. The overall genomic organization of this virus was similar to known picornaviruses. Phylogenetic analysis of the polyprotein demonstrated that the virus was divergent from previously described picornaviruses and appears to belong to the newly proposed picornavirus genus, *Cosavirus*. Based on the analysis discussed here, we propose that this virus represents a new species in the Cosavirus genus, and it has tentatively been named Human Cosavirus E1 (HCoSV-E1).

## Findings

Diarrhea is the third leading infectious cause of death worldwide and causes approximately 2 million deaths each year [1]. Additionally, an estimated 1.4 billion non-fatal episodes occur yearly [2,3]. Importantly, it is estimated that 40% of diarrhea cases are of unknown etiology [4-6]. Motivated by an interest to identify novel or unrecognized viruses associated with diarrhea, we recently developed a mass sequencing strategy to define the spectrum of viruses present in human stool [7]. Using this approach, we describe here the identification of a novel virus in a stool sample collected in 1981 at the Royal Children's Hospital in Melbourne, Australia from a child with acute diarrhea.

Previous testing of this diarrhea specimen for known enteric pathogens using routine enzyme immunoassays (EIA) and culture assays for rotaviruses, adenoviruses, and common bacterial and parasitic pathogens was negative [8]. Additionally, RT-PCR assays for caliciviruses and astroviruses were also negative [8,9], making this sample a good candidate for viral discovery efforts as described [7].

In brief, 200 mg of frozen stool was chipped and then resuspended in 6 volumes of PBS [7]. The sample was centrifuged to pellet particulate matter and the supernatant was then passed through a 0.45 μm filter. RNA was isolated from 100 μL primary stool filtrate using RNA-Bee (Tel-Test, Inc.) according to manufacturer's instructions. Approximately, 100 nanograms of RNA was randomly amplified using the Round AB protocol as previously described [10]. The amplified nucleic acid was cloned into pCR4.0 using the TOPO cloning kit (Invitrogen, Carlsbad, CA), and clones were sequenced using standard Sanger chemistry [7]. High quality sequences were compared to the GenBank nr database by BLASTx and one 395 bp sequence read was identified in this sample that had only 55% identity at the amino acid level to its top hit, the VP3 protein of Theiler's-like virus, a murine picornavirus in the genus cardiovirus.

Picornaviruses are non-enveloped viruses with a single stranded positive-sense RNA genome that encodes a single polyprotein [11]. The genomes range in size from approximately 7 kb to 8.5 kb in length, are polyadenylated, and have 5' and 3' non-translated regions. The 5'-non-translated regions of picornaviruses are highly structured and contain an internal ribosome entry site (IRES) that directs translation of the RNA by internal ribosome binding [11]. The 3'-non-translated region also contains a secondary structure, including a pseudoknot, that has been implicated in controlling viral RNA synthesis [11]. Recently, Kapoor et al identified multiple novel related picornaviruses which they propose belong to a new genus, cosavirus. These viruses were found in the stools of both healthy children and those with acute flaccid paralysis in Pakistan and Afghanistan [12]. Additionally, 1 stool from a 64 year old woman in Scotland was found to be positive for Human Cosavirus A. Other picornaviruses have also been found in stool such as enteroviruses, polio, and aichi virus [11,13].

Using a combination of direct Sanger sequencing, RT-PCR, 5' and 3' random amplification of cDNA ends (RACE), and 454 sequencing performed on RNA isolated from the stool sample, a 6562 bp contig [GenBank: FJ555055] containing the entire predicted polyprotein and the 3' untranslated region to the poly A tail was generated. For these sequencing experiments, the stool filtrate was proteinase K and DNAse treated prior to RNA extraction. RT-PCR and 3'RACE reactions were performed using SuperScript III and Platinum Taq (Invitrogen One-Step RT-PCR). For 5'RACE reactions cDNA was generated with Stratascript (Stratagene) and amplified with Accuprime Taq (Invitrogen). The initial assembly was confirmed by sequencing a series of four overlapping RT-products to give 2.7× coverage. All amplicons were cloned into pCR4 (Invitrogen) and sequenced using standard sequencing technology. Despite repeated efforts, we were unable to obtain additional sequence at the 5' end, presumably due to the presence of RNA secondary structures. Even performing 5' RACE reactions at 65°C or 70°C with multiple high temperature reverse transcriptases (Monsterscript [Epicentre Biotechnologies], rTth [Applied Biosystems], and Thermoscript [Invitrogen]) did not extend the contig further in the 5' direction.

**Figure 1:**
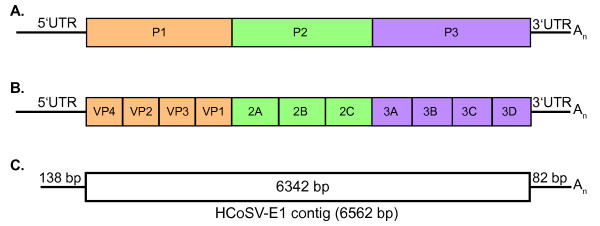
**Genomic organization of *Cosavirus***. Schematic of initial protein products P1, P2, and P3 (A). Schematic of processed polyprotein (B). Representation of sequence obtained from Human Cosavirus-E1 (C).

**Figure 2:**
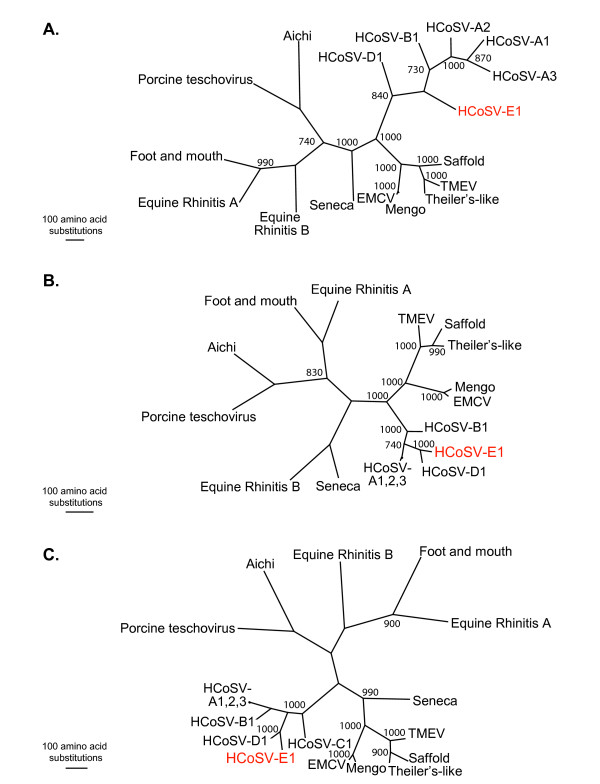
**Phylogenetic Analysis of HCoSV-E1**. Multiple sequence alignments were generated with HCoSV-E1 P1 (A), P2 (B), and P3 (C) sequences and the corresponding regions of known picornaviruses using ClustalX. PAUP was used to generate phylogenetic trees and bootstrap values (> 700) from 1,000 replicates are shown.

Analysis of the contig sequence showed that this virus has a genomic organization similar to other picornaviruses (figure 1). Using Pfam [14], conserved motifs characteristic of picornaviruses were found to be present, including two picornavirus capsid proteins, RNA helicase, 3C cysteine protease, and RNA dependent RNA polymerase. Predicted polyprotein cleavage sites were identified by scanning for conserved amino acids characteristic for cleavage sites [GenBank: FJ555055] as described [15]. We performed phylogenetic analysis on each of the three coding regions: P1 (Figure 2A), P2 (Figure 2B) and P3 (Figure 2C). Protein sequences associated with the following reference virus genomes were obtained from GenBank: Equine Rhinitis A virus (NP_653075.1), Foot-and-mouth-type-O (NP_658990.1), Equine Rhinitis B virus (NP_653077.1), Theiler's-like virus of rats (BAC58035.1), Saffold virus (YP_001210296.1), Theiler murine encephalomyelitis (AAA47929.1), Mengo virus (AAA46547.1), Encephalomyocarditis virus (CAA60776.1), Seneca valley virus (DQ641257), Aichi virus (NP_047200.1), and Porcine teschovirus (NP_653143.1). Human cosavirus sequences (FJ4388825-FJ438908 and FJ442991-FJ442995) were kindly provided by E. Delwart. Multiple sequence alignments were performed using ClustalX (1.83). The amino acid alignments generated by ClustalX were input into PAUP [16], and maximum parsimony analysis was performed using the default settings with 1,000 replicates.

Phylogenetic analysis demonstrated that this virus sequence is highly divergent from previously described picornaviruses and is most closely related to viruses in the newly reported genus cosavirus (Figure 2) [12]. According to the Picornavirus study group [17] members of a genus should share > 40%, > 40% and > 50% amino acid identity in P1, P2 and P3 genome regions respectively. For all picornavirus genera except apthovirus, species are defined as sharing > 70% amino acid identity in P1 and > 70% amino acid identity in 2C and 3CD [18]. Sequences from the 4 previously described cosavirus species share 48–55% amino acid identity in the P1 region to each other and 63–72% identity in the 3D [12]. This virus had 51% amino acid identity to the P1 region, 88% amino acid identity to 2C, and 77% amino acid identity to 3CD of HCoSV-D1, its closest relative based on phylogenetic analysis of the entire polyprotein (data not shown). Given that this virus does not meet all criteria for inclusion in the existing cosavirus species, we propose that this virus be considered a new species within the cosavirus genus. Therefore we have tentatively named this virus Human Cosavirus E1 (HCoSV-E1).

A subset of viruses in the family *Picornaviridae*, members of the genera Cardiovirus, Apthovirus, Erbovirus, Kobuvirus, Teschovirus and the proposed genera Sapelovirus and Senecavirus [11,19,20], encode a leader protein (L) at the N terminus of the polyprotein. In addition, cardioviruses also encode for a L* protein, a protein that is initiated from an alternative AUG downstream from the initiation site of the polyprotein. Neither HCoSV-E1 nor the other described members of the proposed genus cosavirus appeared to encode an L or L* protein. [12]

253 pediatric stool specimens sent to the clinical microbiology lab for bacterial culture at the St. Louis Children's Hospital and 143 stool samples from children with acute diarrhea at the Royal Children's Hospital (Melbourne, Australia) were analyzed for the presence of HCoSV-E1 by RT-PCR using primers (LG0053: 5'-GAACTCATGCAACTTACCCAGC-3' and LG0052: 5'-GCCAAGACATGATCCAACGG-3') designed to the 3D region of the genome. None of these samples were positive for the presence of HCoSV-E1. This suggests that the prevalence rate of HCoSV-E1 is more similar to the reported cosavirus prevalence in Scotland (1/1000) than that described in Pakistan [12]. However, obtaining more sequence from the 5'UTR of HCosV-E1, would enable design of more robust screening primers to more comprehensively analyze these cohorts for the presence of viruses closely related to HCosV-E1. Additionally, usage of conserved primers capable of detecting all of the known cosaviruses could potentially reveal the presence of other cosaviruses in these cohorts of stool samples.

At this time the relationship of HCoSV-E1 to diarrhea or other human diseases is unknown. One possibility is that HCoSV-E1 represents a true human pathogen that causes gastroenteritis. Alternatively, it may be a human pathogen that is shed in the stool, but causes extraintestinal disease such as poliovirus. Another possibility is that HCoSV-E1 may be a commensal or symbiotic microbe. Additionally, it is also possible that HCoSV-E1 is a result of dietary ingestion and is not a virus that truly infects or replicates in human cells. Regardless of the clinical role of HCoSV-E1, the identification of HCoSV-E1 in this study further emphasizes the tremendous microbial diversity of the human gut that remains to be discovered and the need for systematic investigations of the human "virome". In addition, future work will focus on defining if HCoSV-E1 is a true human pathogen.

## Competing interests

The authors declare that they have no competing interests.

## Authors' contributions

DW conceived and designed the experiments. LH carried out the experiments and analysis. SF participated in the design and analysis of the experiments. CK contributed samples and edited manuscript. LH and DW wrote the paper.
